# Seismic structure of the 2015 M_w_7.8 Gorkha earthquake revealed by ambient seismic noise and teleseismic surface wave tomography

**DOI:** 10.1038/s41598-024-57713-8

**Published:** 2024-04-04

**Authors:** Ziqiang Lü, Jianshe Lei, Qinghan Kong, Qian Liu, Jingwen Sun

**Affiliations:** 1 College of Mining, Liaoning Technical University, Fuxin, China; 2 Key Laboratory of Crustal Dynamics, National Institute of Natural Hazards, Ministry of Emergency Management of China, Beijing, China

**Keywords:** Geophysics, Seismology

## Abstract

The destructive 2015 M_w_7.8 Gorkha earthquake occurred in the Main Himalayan Thrust due to the collision of the Indian and Asian plates, which provides a unique opportunity to understand the deep dynamic processes and seismogenic mechanisms of strong earthquakes. We construct a regional-scale shear-wave velocity model of the crust and uppermost mantle using ambient seismic noise and teleseismic surface wave at periods of 5–100 s around the Gorkha earthquake region. The new shear-wave velocity model exhibits prominently lateral heterogeneities in the Gorkha earthquake areas. We observe a high-velocity (high-V) zone around the Gorkha main shock in the Main Himalayan Thrust, indicating the existence of a high-strength asperity that sustains the stress accumulating. The aftershocks are primarily located in the low-velocity (low-V) anomalies and enclosed by two high-V anomalies, which appear to act as structural barriers that influence the spread of the aftershocks. Prominent low-Vanomalies from the lower crust to the mantle lithosphere are observed along the north–south trending rifts, suggesting the hot materials upwelling due to the tearing of the northward subducting Indian lithosphere. These observations may indicate that seismic velocity heterogeneity could play an essential role in earthquake initiation and the rupture process.

## Introduction

**Figure 1:**
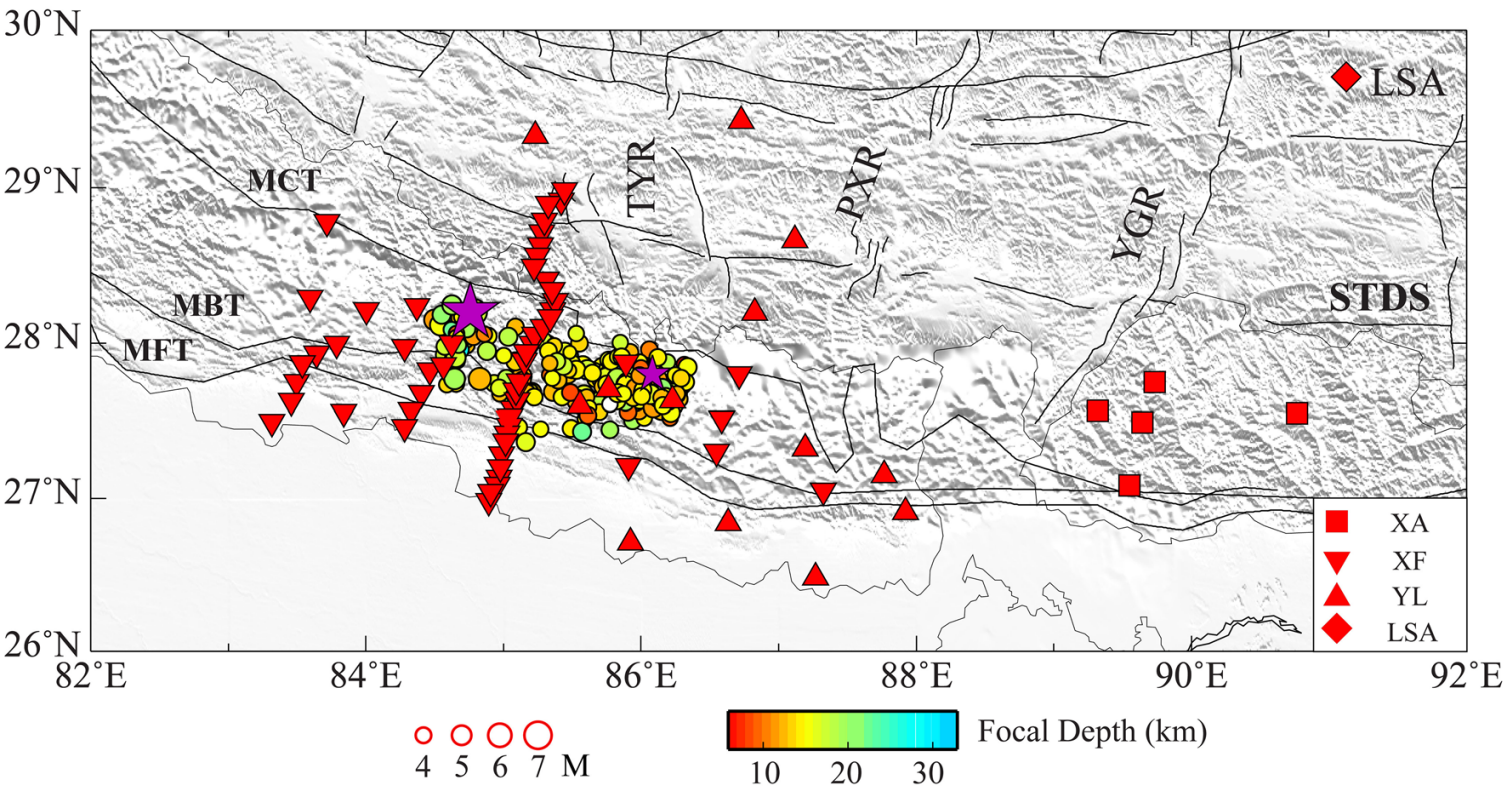
Regional tectonic map with seismic stations and seismicity. The purple stars represent the locations of the Mw7.8 Gorkha mainshock and the Mw7.2 aftershock, respectively. The circles denote the aftershocks of the Gorkha mainshock with different depths and magnitudes. The scales are shown at the bottom. MFT, Main Front Thrust; MBT, Main Boundary Thrust; MCT, Main Central Thrust; STDS, Southern Tibet Detachment System; YGR, Yadong Gulu Rift; PXR, Pumqu Xainza Rift; TYR, Tangre Yumco Rift. Imagery is available from the U.S. Geological Survey (https://lpdaac.usgs.gov/products/srtmgl1v003). Figure made with Generic Mapping Tools^6^ (GMT v.6.4.0: https://www.generic-mapping-tools.org).

The Himalayan orogenic belt, a typical example of collision tectonics, was formed by the continuing interaction of the Indian and Asian plates with a rate of ~ 20 mm/year^1^, and produced frequent earthquake hazards with great casualties and property losses^2^. On 25 April 2015, a reverse-faulting M_W_ 7.8 Gorkha earthquake occurred along the Main Himalayan Thrust (MHT), with over 9,000 deaths and more than 22,000 injured. The Gorkha earthquake showed a typical thrust-fault focal mechanism with a low dip angle^3^, and the rupture propagated approximately 120 km along the strike with a maximum slip of ~ 6 m^4^. The Gorkha mainshock triggered a large number of aftershocks (Fig. 1), including the 12 May 2015 M_w_ 7.3 Kodari aftershock that was initiated near the eastern boundary of the mainshock rupture^5^. Furthermore, several other historical earthquakes occurred in the Himalayan region, including the 1505 M_s_ 8.2 Karnali River earthquake, the 1897 M_s_ 8.0 Kathmandu earthquake, the 1934 M_s_ 8.0 Bihar earthquake, and the 1950 M_s_ 8.5 Assam earthquake^2^. The deep structure of these large earthquakes can provide significant information on the seismogenic mechanisms of strong earthquakes and the evaluation of seismic hazards.

The strong convergence rate of the Indian and Asian plates is absorbed in the Himalayan orogenic belt, leading to a steep topographic gradient and high-stress accumulation^1^. The Himalayan orogenic belt is composed of several complex thrust faults, such as the Main Frontal Thrust (MFT), Main Boundary Thrust (MBT), and Main Central Thrust (MCT). These thrust faults merge into the upper-to-middle crust of the MHT, which caused a series of catastrophic earthquakes along the Himalayan orogenic belt^7^. A series of north–south trending rifts, including Yadong Gulu Rift, Pumqu Xainza Rift, and Tangre Yumco Rift, developed in southern Tibet, which may be related to the east–west extension of the Tibetan plateau^8^. These rifts are close to the rupture zone of the Gorkha earthquake and could have influenced the rupture process.

The crustal structure and the potential localized deformation of the Tibetan plateau are still subject to debate. Several models have been established to explain the dynamic responses to collision and the mechanism of lithospheric deformation, which includes the rigid block extrusion model^9^, the thin viscous sheet model^10^, and the crustal flow model^11^. Previous seismic tomography showed that the Indian plate has subducted to the northern Tibetan plateau^12–15^. The subducted frontier may have reached beneath the Qiangtang block and has subducted to 200 km depth based on the receiver function analyses^16–18^. Pn and Pg wave tomography revealed that the Gorkha earthquake was located in a high-velocity zone and crustal tearing on both sides of the rupture zone of the mainshock^19,20^. However, considering the factors that these studies are either low lateral resolution or two-dimensional images, which limits our knowledge of the dynamics of the subducting Indian plate and seismogenic mechanisms of strong earthquakes.

In this study, we construct a new 3-D crust and upper mantle shear-wave velocity model around the 2015 MW 7.8 Gorkha earthquake region using ambient seismic noise and teleseismic surface wave tomography. The small-scale ambient seismic noise can well constrain the seismogenic structure of the Gorkha earthquake and the teleseismic surface wave can provide additional information on the deep dynamic processes. Our tomographic model reveals a significant lateral seismic velocity contrast from the crust to the upper mantle along the strike of the orogenic belt. The Gorkha main shock occurred in a high-velocity zone on the Main Himalayan Thrust and the distribution of the aftershocks was associated with the seismic velocity anomalies of the seismogenic region. The new model provides more structural constraints on the generation of strong earthquakes and sheds light on dynamic processes responsible for the uplift mechanism of the Himalayan orogenic belt.

## Rayleigh-wave phase velocity maps

The ray-path coverage maps of Rayleigh-wave phase velocity at various periods are shown in Fig. S4. Generally, the ray-path coverage is quite well for most of the study region, especially for the Gorkha earthquake region. To determine the spatial resolution scale, we carry out a series of checkerboard resolution tests to evaluate the recovering capabilities of the dispersion data (Fig. S5). The input models are conducted with alternating ± 8% velocity perturbation with different anomaly sizes at different periods. The perturbation pattern and amplitude are generally recovered in most of the study area at periods of 10–80 s with the anomaly size of 1.0° × 1.0°, whereas the pattern and amplitude can be recovered with the anomaly size of 1.5° × 1.5° at periods of 100 s due to the long period dispersion reduction.

We construct 2-D Rayleigh-wave phase velocity structures at different periods (Fig. S6). The fundamental Rayleigh wave phase velocities at different periods are primarily sensitive to the shear velocity at various depths (Fig. S7). The short period phase velocities (< 20 s) are very sensitive to the shallow geological features (~ 5–30 km depths), which are spatially correlated with correlated with the surface tectonic features. The Himalayan orogenic belt mainly shows high-velocity (high-V) anomalies, and the north–south trending rifts, such as Tangre Yumco rift (TYR), Pumqu Xainza rift (PXR), and Yadong Gulu rift (YGR) display low-velocity (low-V) anomalies. The phase velocities at the period of 40–60 s roughly reflect velocity anomalies between the lower crust and uppermost mantle (~ 40–100 km depths), and the amplitude of the low-V anomalies beneath the north–south trending rifts decreases with depth. The phase velocity maps at periods of 80–100 s are more sensitive to the depths of ~ 110–180 km, and the low-V anomalies appear to extend from the Himalayan orogenic belt to the north–south trending rifts.

## Shear-wave velocity structure

**Figure 2:**
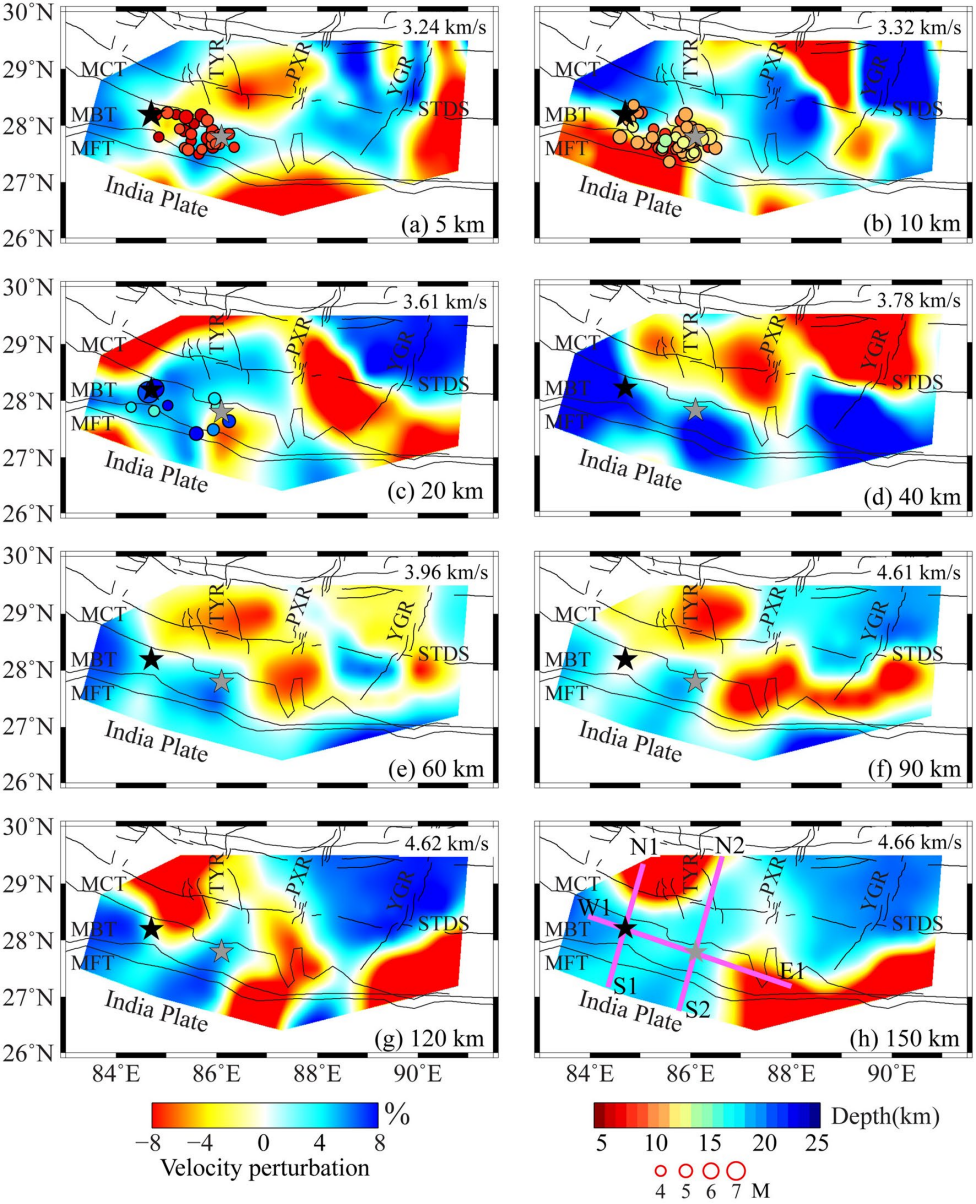
Shear-wave velocity model at different depths. Black and gray stars represent the M_W_ 7.8 Gorkha mainshock and the M_W_ 7.2 aftershock, respectively. Circles denote aftershocks of the Gorkha mainshock, and the size and color of which denote earthquake magnitude and focal depth. The scales for velocity perturbation, earthquake magnitude, and focal depths are shown at the bottom. The mean velocity is shown at the top-right corner of each map. The pink lines in (**h**) show the locations of cross sections in Fig. 3. See the descriptions in Fig. 1 for other labels.

**Figure 3:**
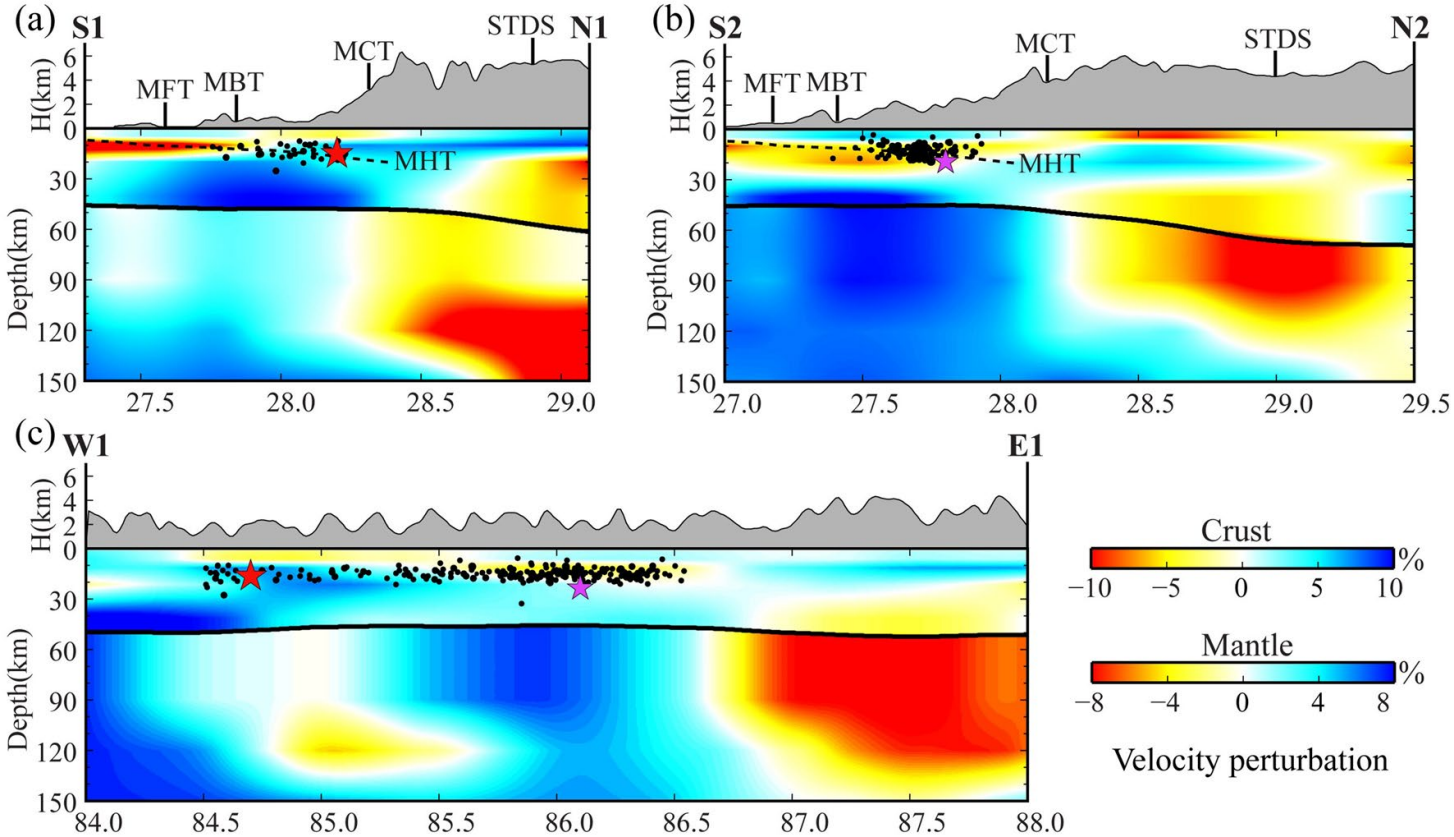
The vertical profiles of the shear-wave velocity model. See Fig. 2h for the profile locations. The red and purple stars denote the M_W_ 7.8 Gorkha mainshock and the M_W_ 7.2 aftershock, respectively. The black dots denote the aftershocks within the range of 60 km off the profile. Topography is depicted above each profile, while the black lines denote the Moho depths obtained in the study. The color scales are shown different velocity perturbations for the crust and mantle, respectively.

The crust and upper mantle structures are more complicated as the result of the subduction of the Indian plate and the upwelling of hot and wet materials (Fig. 2). At shallow depths (5–10 km), low-V anomalies mainly appear under the Indian plain with thick sediments and pronounced low-V anomalies appear to the south of MBT at depth of 10 km. These characteristics are closely related to the surface tectonic features. From the upper crust to the middle crust (20–40 km depths), the Himalayan orogenic belt is gradually imaged with strong high-V anomalies, and the north–south trending rifts display pronounced low-V anomalies at a depth of 40 km. At the crust-mantle boundary (60 km depth), the low-V anomalies are present in the north–south trending rifts and appear to be separated by thin high-V anomalies. In the upper mantle (90–120 km depths), the PXR and YGR area show high-V anomalies and these features gradually strengthen with depths, whereas the TYR area appears low-V anomalies from the middle crust to the upper mantle. We use different perturbation scales for visualizing vertical velocity profiles due to the crust and mantle have different scales of heterogeneity and velocity variations, which are crucial for understanding geodynamic processes and seismic hazards. The significant lateral seismic velocity contrast in the upper mantle across the MCT, including the strong high-V anomalies to the south of the MCT, and prominent low-V anomalies to the north of the MCT, respectively (Fig. 3a and b). The Moho depth obtained in the present study shows that the Moho varies with shallower depth in the south and deeper depth in the north, suggesting that the Indian plate is subducting beneath the Eurasian plate. The shear-wave velocity structure beneath the Gorkha earthquake region shows strong seismic heterogeneities (Figs. 2 and 3). The M_W_ 7.8 main shock is located in the high-V anomalies and the largest aftershock occurred in the low- to high-velocity transition zone. Most aftershocks are located in the low-V anomalies and extend southeastward from the main shock to the largest aftershock.

## Discussion

The collision of the Indian plate and the Eurasian plate caused north–south crustal shortening and thickening of the lithosphere along the Himalayan orogenic belt. The crustal shortening rates are about 20.5 mm/year in western Nepal and 17.8 mm/yr in eastern Nepal, respectively^21^. In addition, there are several north–south trending rifts and normal faults in southern Tibet, corresponding to the Cenozoic east–west extension of the plateau^22^. These phenomena implicate that north–south continental collision and the east–west extension in southern Tibet is a complex tectonic process.

Compared with the previous models using ambient seismic noise tomography^23,24^, our present tomographic model shows some similar features (Fig. 3). For example, there are obvious high-V anomalies beneath the Gorkha main shock in the crust, whereas relatively weaker velocities are revealed beneath the Bhutan Himalaya, and low-V anomalies are observed beneath the Indian plain above 20 km depths. However, our model also reveals some new features. For instance, the northward dipping low-V anomalies are observed along the MHT, and significant low-V anomalies are observed in the north of the MCT. These observations are also supported by the receiver function image and finite frequency tomography^25,26^. These significant improvements are made as a result of utilizing much more high-quality data from multiple networks. Discrepancies exist in these tomographic models due to diverse methodologies and data sets.

By integrating the ambient seismic noise and teleseismic surface wave, a new crust and upper mantle shear-wave velocity model is constructed, which presents new seismic constraints on the underlying dynamics of the collision between the Indian plate and Eurasian plate. Our new model exhibits the significant high/low seismic velocity contrast in the upper mantle across the MCT. Previous large-regional tomographic studies revealed high-V anomalies as the subducting Indian lithosphere mantle along the Himalayan orogenic belt^12,25^, however, the above models are not enough to constrain the boundary of the seismic velocity contrast in the Gorkha earthquake region. In this study, the high-V anomalies in the upper mantle are imaged in the south of the MCT, whereas the low-V anomalies are observed in the north of the MCT (Fig. 3). These features imply the MCT is a significant boundary for potential lithospheric deformation. The relatively thin crust and high Pn velocity are also observed to the south of the MCT^19,27^, which are typically in line with the observed high-velocity anomalies. Instead, the previous magnetotelluric array study reveals an apparently decreased resistivity anomaly around the northern MCT^28^, corresponding with the observed low-V anomalies in this region. We propose that the high-velocity anomalies represent the subducting Indian lithosphere that may be torn near the MCT. In this sense, the tearing of the Indian lithospheric mantle may form a pathway along the MCT, which could induce the local mantle upwelling, contributing to seismic velocity reduction. Additionally, the material from the upwelling likely moves upward towards the crust, as indicated by the relatively low velocities in the middle and lower crust beneath the north–south trending rifts. Therefore, the MCT represents a major boundary that influences the deformation of the subducting Indian lithosphere and the evolution of the north–south trending rifts in the study region.

**Figure 4:**
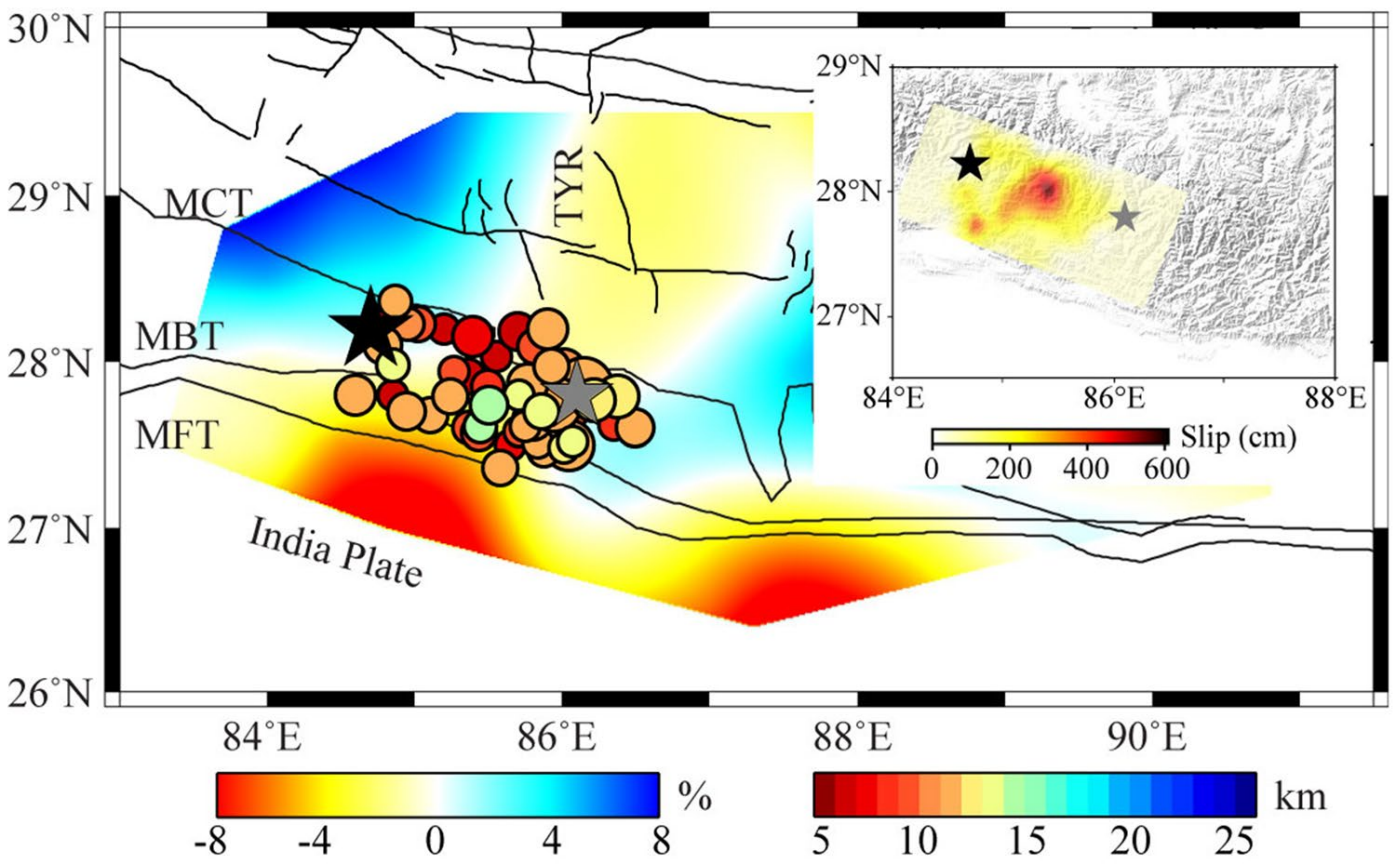
The background is the average shear-wave velocity above 10 km. The inset shows the slip model of the Gorkha earthquake from Wang et al.^4^. Other symbols are the same as in Fig. 2. Imagery is available from the U.S. Geological Survey (https://lpdaac.usgs.gov/products/srtmgl1v003). Figure made with Generic Mapping Tools^6^ (GMT v.6.4.0: https://www.generic-mapping-tools.org).

The seismic structural heterogeneity plays a crucial role in the occurrence of large earthquakes^29–32^. The behavior of the MHT has been known to trigger numerous significant earthquakes in the Himalayan orogenic belt. The property of the MHT has been identified from a variety of geophysical methods that relatively low resolution at depths, such as shearing anisotropic fault^33^, intense seismic activity^34^, and high electrical conductivity^35^. These characteristics imply the MHT could be a weak zone with seismic velocity reduction, caused by the release of trapped water from underthrusting sediments^26^. In this study, we observe pronounced low-V anomalies in the upper crust extending from the subducting front to the MCT, indicating the presence of the MHT. The Gorkha main shock was located in the high-V anomalies at the northern of the MHT and the aftershock activities were mainly distributed along the MHT and became deeper to the north (Fig. 3a and b). The distribution of the shallow crustal velocity structure is roughly consistent with the radiated energy of the Gorkha earthquake (Fig. 4). The source rupture process showed a predominantly unilateral rupture from the main shock to the southeast with a peak slip of ~ 6 m^4,36^. The initially small rupture corresponds to the observed high-V anomalies zone beneath the Gorkha main shock and the following large rupture generally correlates with the low-V anomalies zone beneath the aftershocks areas (Fig. 4 and Fig. S8). Here, we suggest that the high-V anomalies indicate the presence of an asperity with high-strength rocks, promoting the strain accumulated continually in the Gorkha main shock nucleation stage. Moreover, the upwelling of hot materials beneath the north–south trending rift could be another contributing factor to the occurrence of the Gorkha main shock. The upwelling of hot materials extends to the upper crust near the Gorkha main shock and may reduce the effective normal stress on the fault planes, which could be more conducive to the occurrence of the Gorkha earthquake. The aftershock activities were mainly located between the Gorkha main shock and its largest aftershock. Interestingly, we observe two high-V zones in the upper crust in the western segment of the Gorkha main shock and the eastern segment of the largest aftershock, respectively (Fig. 4). The two high-V zones seem to be the structural barriers that control the extension of the aftershocks. Following our observations of the spatial correlation between seismic velocity anomaly and the distribution of the earthquakes, the morphology of the MHT and the structural heterogeneities of the seismogenic area likely influence the rupture process in different convergent zones between the Indian and Eurasian plates collision.

## Conclusions

The M_w_7.8 Gorkha earthquake occurred due to the continental collision between the Indian and Eurasian plates, making it one of the most seismically hazardous regions. In this study, we present a 3-D shear-wave velocity model from the crust to the upper mantle using ambient seismic noise and teleseismic surface wave tomography. The new tomographic model reveals a high-V zone beneath the Gorkha main shock, suggesting the high strength asperity plays a crucial role in the generation of large earthquakes. The aftershocks are mainly distributed in the low-V anomalies with large rupture, and enclosed by two high-V anomalies, which seem to be the structural barriers that control the extension of the aftershocks. Moreover, the high-V anomalies and the low-V anomalies in the upper mantle are imaged to the south and north of the MCT, respectively, implying the MCT is a lithospheric boundary in the study region. The new findings demonstrate that structural heterogeneity plays a crucial role in the strong earthquake nucleation and deep dynamic processes along the Himalayan orogenic belt.

## Data and method

With the purpose of probing the seismogenic mechanism of the Gorkha earthquake, we collect the Rayleigh-wave dispersion data from seismic ambient noise and teleseismic surface wave using the vertical-component waveforms between the stations. The relatively short period dispersions at periods from 5 to 30 s were obtained from ambient noise interferometry and the teleseismic surface wave extracted the dispersion data within a range of 18 to 100 s. The data of this study are mainly from 4 networks, including 3 temporary seismic observation networks (XA, XF, YL)^26,37,38^, and 1 permanent station (LSA), a total of 81 stations (Fig. 1). According to the continuity of ambient noise data, the continuous waveform data of XF network were selected from October 2002 to December 2003, YL network continuous waveform data were selected from October 2001 to April 2003, XA network data were selected from January 2002 to December 2002, and LSA station data were selected from October 2001 to December 2003. Given the presence of the permanent and stable record of the LSA station, we can obtain dense interstation cross-correlations throughout the study region.

Generally, in order to improve the signal-to-noise ratio (SNR) of empirical Green’s functions (EGFs), a series of preprocessing steps should be applied to the waveform data^39^. We cut the continuous data to one-day length with a band-pass filter of 1.25–50 s, removed mean and trend, adjusted the daily length data to a sample rate of 1 Hz to improve computational efficiency, and applied normalization in the time domain and spectral whitening in the frequency domain^40^. Then, the SNR should be above 5 to enhance the accuracy of phase velocity measurements. Here, the SNR is defined as the ratio of the maximum amplitude of the signal window to the mean envelope amplitude of the 150 s long noise window. In order to guarantee the data has a high SNR and produces reliable tomographic images, we finally choose the stacked cross-correlation functions over 150 days for the phase velocity dispersion curve extraction. The fundamental phase velocity dispersion data of the Rayleigh wave were obtained from the EGFs using the image analysis method. This method filters the EGFs within a certain frequency band and transforms time-period image to velocity-period image, which can effectively identify the fundamental dispersion curve^41^.

Due to the dispersion data of the EGFs being mainly sensitive to crustal structures, we proceeded to extract the dispersion data at longer periods with the teleseismic surface wave in order to better constrain deeper structures. The two-station analysis was used to measure the dispersion from the teleseismic surface wave within the periods of 18 to 100 s^41^. A total of 1638 high-quality interstation phase velocity dispersions were obtained from teleseismic events (Fig. S1). The dispersions have been selected following the following criteria, the earthquake magnitude is greater than 5.5, with a minimum epicentral distance greater than 10°. There are two parameters (α and β) in the two-station analysis. α is defined as the azimuthal difference between the earthquake to the two stations, and β is defined as the azimuthal difference between the earthquake to the station and the two stations. For more details, refer to Yao et al.^41^. We define α and β in the two-station analysis are less than 3° and 7°, respectively, which keeps the earthquakes and station pairs almost on the same great circle path. Figure S1 shows the epicentral distribution of 246 earthquakes used in this study. Most earthquakes were mainly concentrated on the entire eastern hemisphere, on the whole, the events have better azimuth coverage.

Considering the dispersion data from the EGFs and two-station analysis both cover the period between 18 and 30 s, we combine the overlapped period band by taking an average between the EGFs-based and two-station-based measurements. The averaging scheme can address the issue of a sharp decrease in the number of EGFs as the period increases and improve the reliability of the two-station analysis at short periods^42^. We also removed data that deviated by more than ± 0.2 km/s from the average dispersion curve to ensure data quality. All dispersion data and its standard deviations at different periods are shown in Fig. S2. The standard deviations from EGFs are smaller than the standard deviations from the two-station analysis, indicating that ambient seismic noise can provide accurate measurements of phase velocity at relatively short periods.

The 2-D distributions of Rayleigh-wave phase velocities at different periods are constructed using the continuous regionalization and the generalized inversion scheme developed by Yao et al.^43^. We set the cell size for inversion to be 0.5° × 0.5° and set the correlation length that determines the smoothness of phase maps to be 100 km at 5–80 s and 150 km at 100 s, respectively. The 3-D model is constructed based on the Neighborhood Algorithm, which is capable of identifying regions with good data fitting and deriving the most optimal model^42^. Firstly, we invert the 1-D shear wave velocity structure using Rayleigh wave dispersion data at each grid point. We use the Neighbourhood Algorithm (NA) method to search for the best-fitting model from different random 1-D shear wave velocity models. The initial shear wave velocity model refers to previous studies^44^. The initial model is constrained by 15 parameters, including the Moho depth and shear-wave velocities in six crustal layers and eight upper mantle layers. For each crust layer, we calculate *Vp* and *ρ* based on *Vs* using the empirical relations between elastic wave speeds and density in the Earth’s crust^45^. For each upper mantle layer, *Vp* and *ρ* are taken directly from the *ak*135 model^46^. The reference Moho depth of each grid point is inferred from receiver functions^47^. We allow the Moho depth to vary within a range of ± 5 km from the reference Moho depth, and the thickness of the lower crust and uppermost mantle layer also vary accordingly. Figure S3 illustrates an example of shear-wave velocity inversion, demonstrating that the resulting inverted shear-wave velocity models from initial models display a high degree of consistency, indicating the stability of the inversion process. Then, the inverted shear-wave velocity model of the crust and upper mantle at each grid point is interpolated at corresponding depths to construct the final three-dimensional shear-wave velocity model beneath the Gorkha earthquake region.

### Supplementary Information

Supplementary Figures.

## Data Availability

The seismograms used in this study were downloaded freely from the Incorporated Research Institutions for Seismology Data Center (https://ds.iris.edu/ds/).
