# Supplementary material for: Seismic structure of the 2015 Mw7.8 Gorkha earthquake revealed by ambient seismic noise and teleseismic surface wave tomography
Source: Sci Rep. 2024 Apr 4;14:7921. doi: 10.1038/s41598-024-57713-8 (PMC10995148; doi:10.1038/s41598-024-57713-8)
Supplement: Supplementary file 1 — Supplementary Figures. [file 41598_2024_57713_MOESM1_ESM.pdf]

Supplementary Materials

Seismic structure of the 2015 M_w7.8 Gorkha earthquake revealed by ambient seismic noise and teleseismic surface wave tomography

Ziqiang Lü[1,*], Jianshe Lei[2], Qinghan Kong[1], Qian Liu[1], Jingwen Sun[1]

[1]*College of Mining, Liaoning Technical University, Fuxin, China*

[2]*Key Laboratory of Crustal Dynamics, National Institute of Natural Hazards, Ministry of Emergency Management of China, Beijing, China*

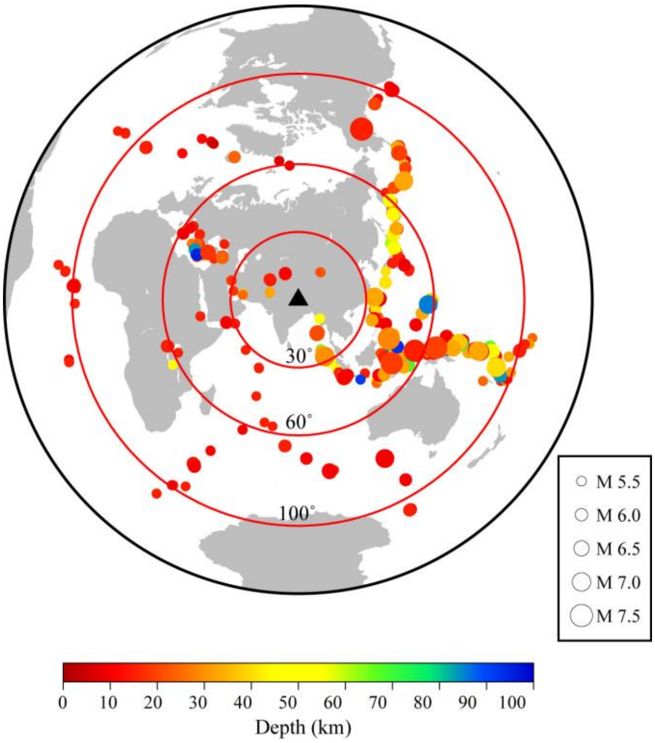

Supplemental Figure 1. The distribution of the teleseismic events used in the two-station analysis. The dots show the locations of teleseismic events with different depths and magnitudes, and the balck triangle shows the center of the study area.

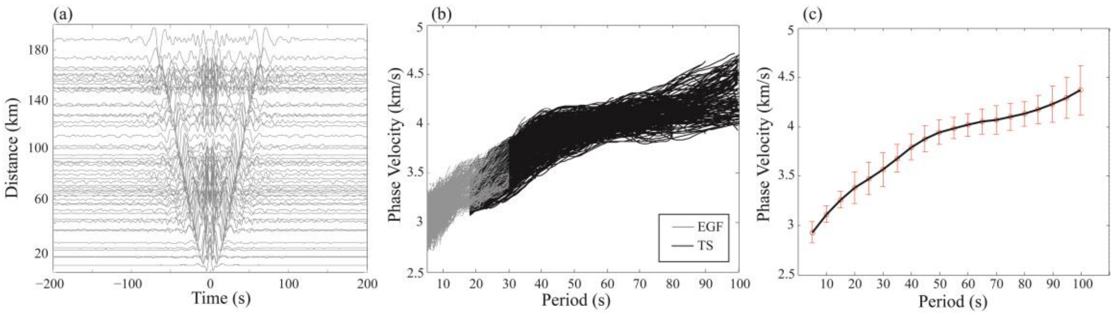

Supplemental Figure 2. (a) Examples of cross-correlation functions derived from ambient noise data. (b) The total dispersion curves from the EGFs and two-station analysis at different periods. (c) The average phase velocity dispersion curve and its standard deviations at different periods. The black line and red error bars in (b) represent the average phase velocity and corresponding standard deviations at different periods.

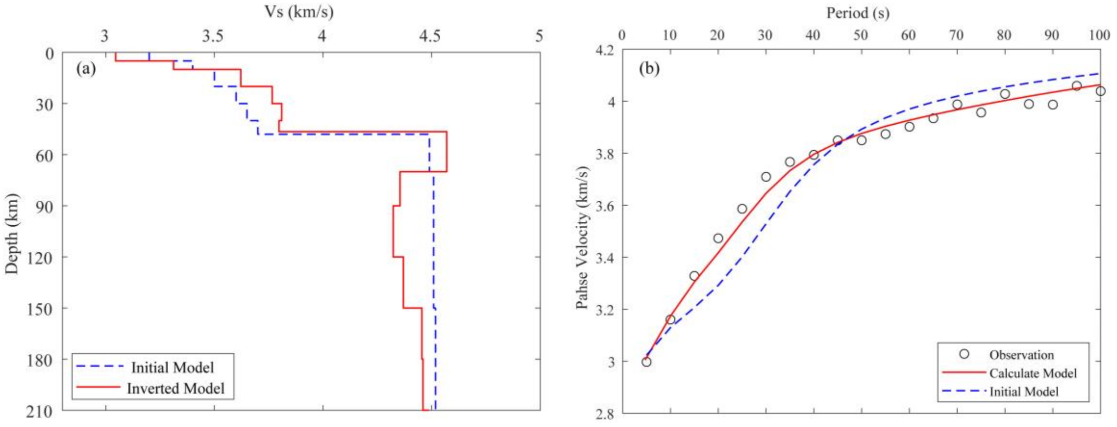

Supplemental Figure 3. Example of the shear-wave velocity inversion at the grid (87 °E, 28 °N) in the center of the study region. (a) Comparison of the initial shear-wave velocity model (blue dash line) and the inverted shear-wave velocity model (red line). (b) Observed and inverted Rayleigh-wave phase velocity dispersions. The red line represents the inverted Rayleigh-wave phase velocity dispersions based on the inverted shear-wave velocity model. The blue dash line is the theoretical dispersions calculated from the initial shear wave speed model.

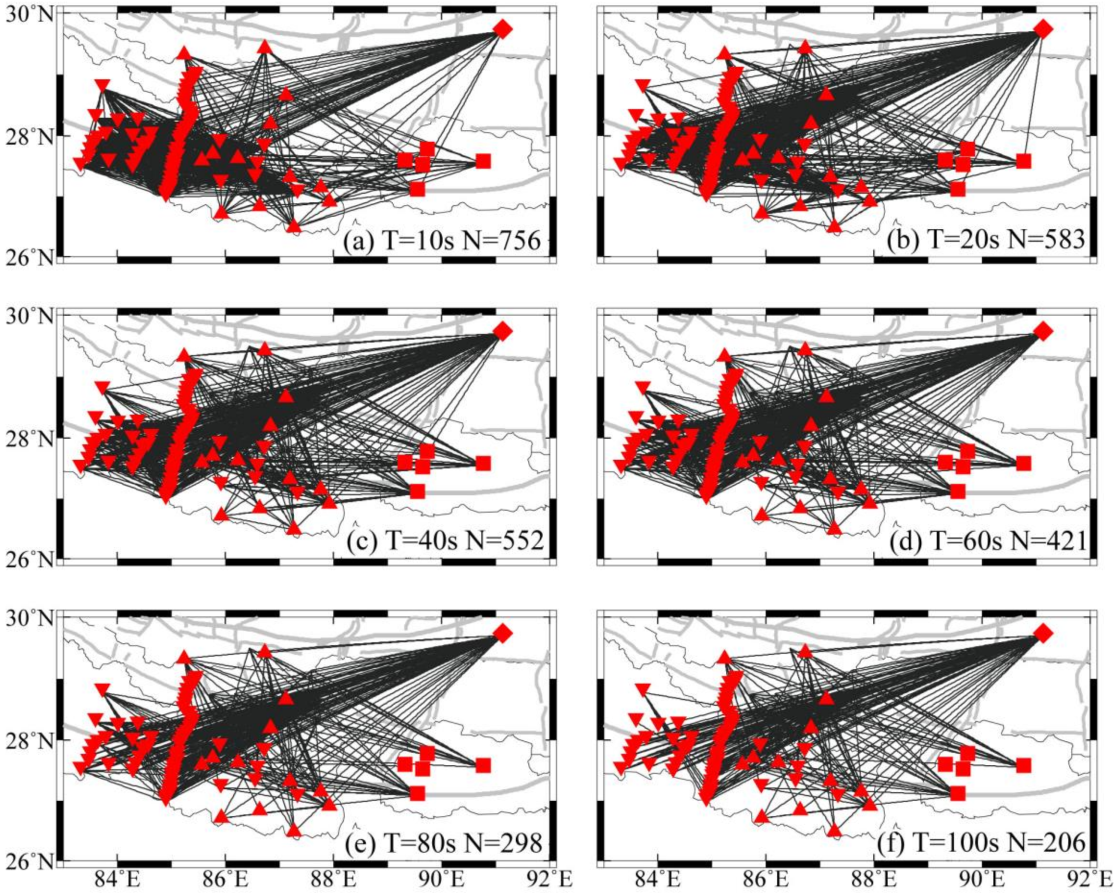

Supplemental Figure 4. Ray-paths (black lines) for Rayleigh-wave phase velocity at different periods. Red triangles denote the used seismic stations and the number of ray paths is shown at each period.

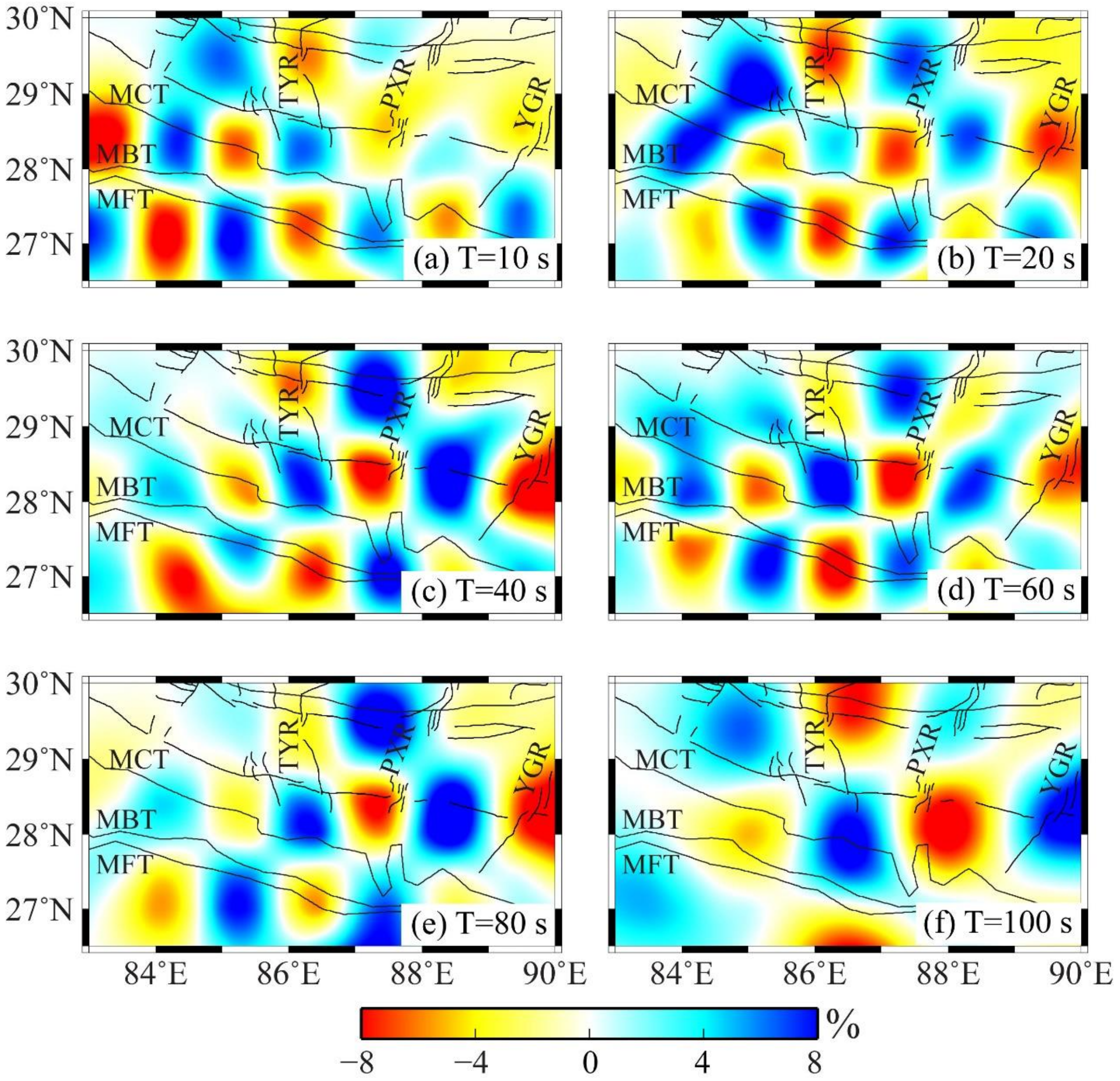

Supplemental Figure 5. Results of checkerboard resolution tests at different periods. The recovered models used a 1.0 °×1.0 ° anomaly size from the period of 10 to 80 s (a-e), whereas the recovered models used a 1.5 °×1.5 ° anomaly size at the period of 100 s (f). Red and blue colors denote low and high-velocity anomalies, the scale of which is shown at the bottom.

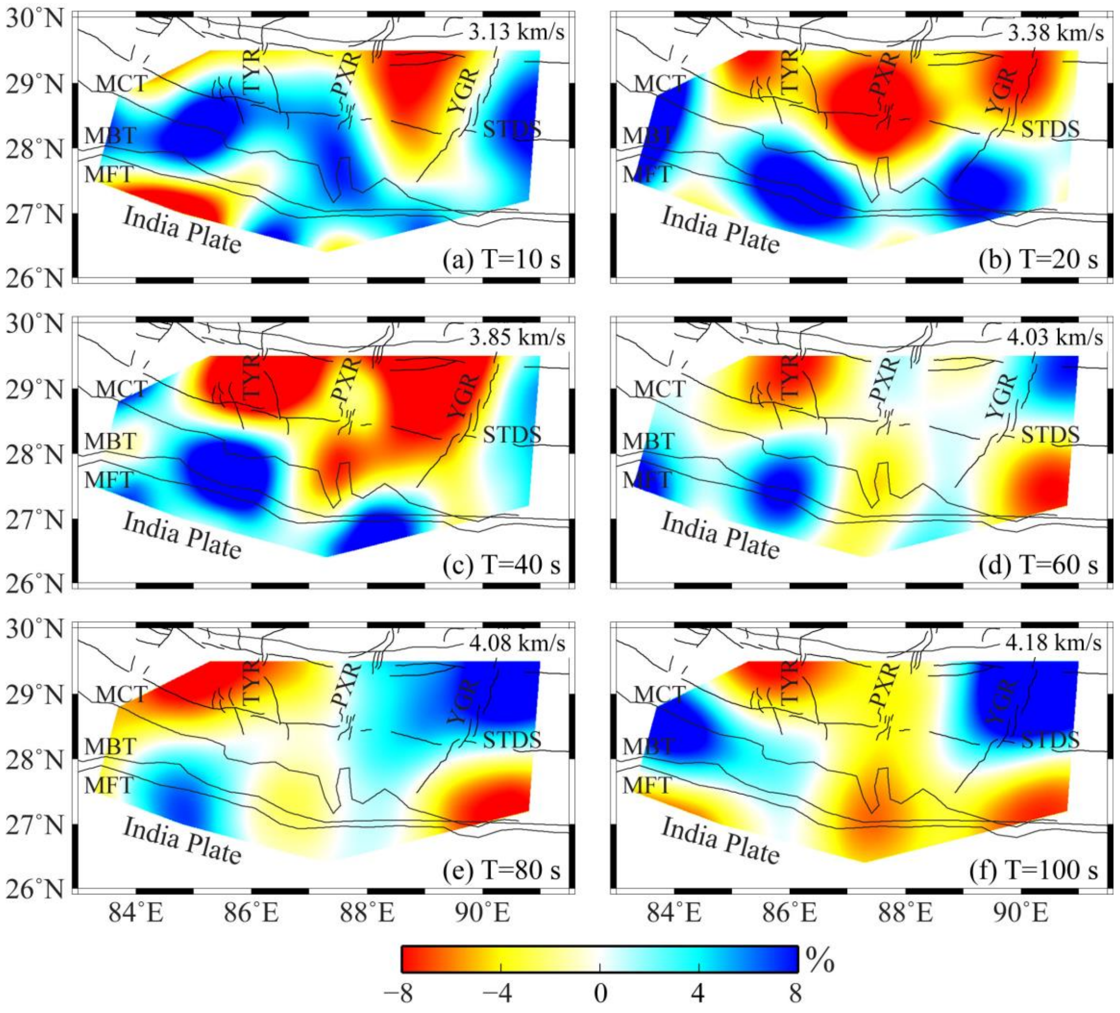

Supplemental Figure 6. Rayleigh-wave phase velocity perturbations at different periods. Red and blue colors denote low and high-velocity anomalies, the scale of which is shown at the bottom. The mean velocity is shown at the top-right corner of each map.

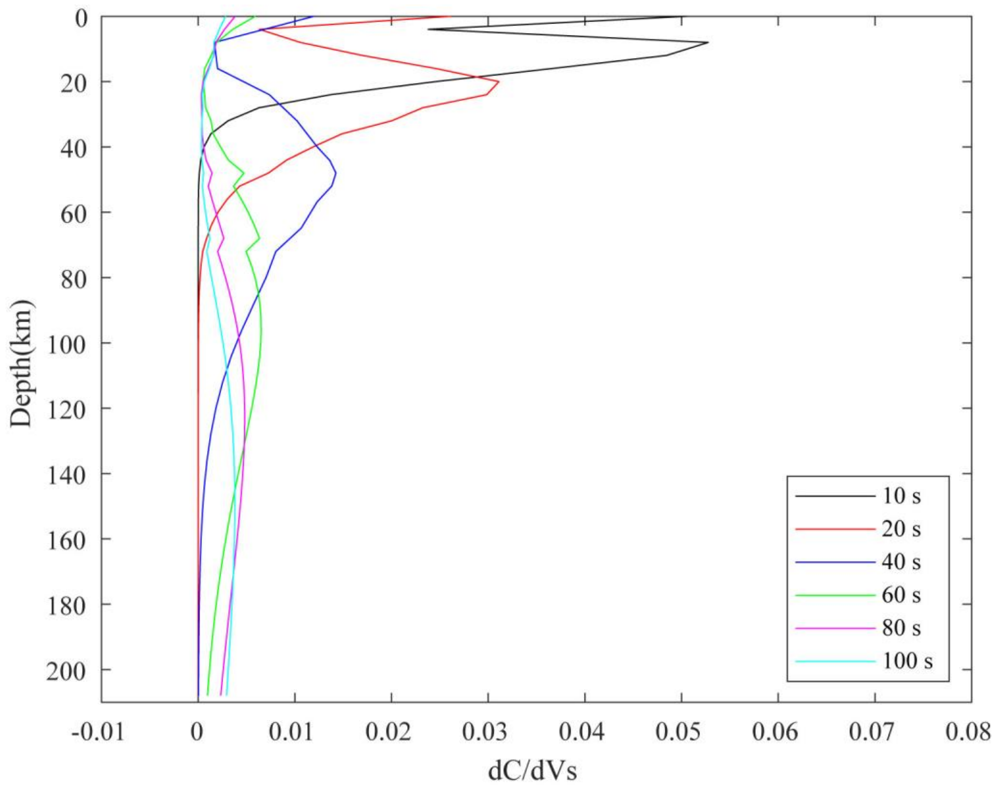

Supplemental Figure 7. Depth sensitivity kernels of Rayleigh-wave phase velocity at different periods.

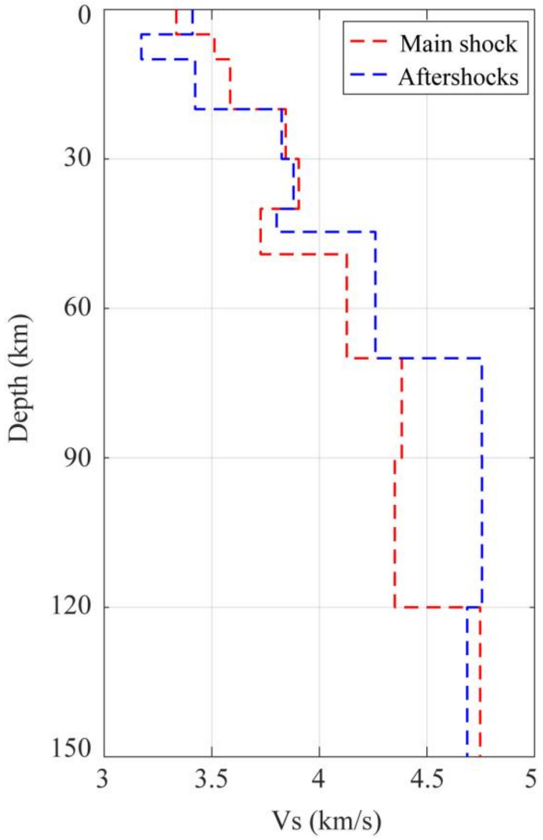

Supplemental Figure 8. Comparison of one-dimensional velocity profile beneath the main shock and aftershocks areas.
